# Malignant Cerebellar Edema Subsequent to Accidental Prescription Opioid Intoxication in Children

**DOI:** 10.3389/fneur.2017.00362

**Published:** 2017-07-25

**Authors:** Daniel Duran, Robert D. Messina, Lauren A. Beslow, Julio D. Montejo, Jason K. Karimy, Charuta Gavankar Furey, Alison D. Sheridan, Gordon Sze, Yanki Yarman, Michael L. DiLuna, Kristopher T. Kahle

**Affiliations:** ^1^Department of Neurosurgery, Yale School of Medicine, New Haven, CT, United States; ^2^Department of Radiology and Biomedical Imaging, Yale School of Medicine, New Haven, CT, United States; ^3^Division of Neurology, Children’s Hospital of Philadelphia, Philadelphia, PA, United States; ^4^Department of Neurology, Perelman School of Medicine at the University of Pennsylvania, Philadelphia, PA, United States; ^5^Yale School of Medicine, New Haven, CT, United States; ^6^Department of Pediatrics, Yale School of Medicine, New Haven, CT, United States; ^7^Department of Cellular and Molecular Physiology, Yale School of Medicine, New Haven, CT, United States

**Keywords:** cerebellar edema, opioid intoxication, pediatric critical care, opiates, suboccipital craniectomy

## Abstract

We present two recent cases of toddlers who developed malignant cerebellar edema subsequent to accidental ingestion of prescription opioids. Both children presented acute neurological decline, hydrocephalus, and tonsillar herniation requiring emergent ventricular drain placement, suboccipital craniectomy, and partial cerebellectomy. Together with several other reports, these cases suggest the existence of an uncommon yet severe syndrome of acute opioid-induced malignant cerebellar edema. We hypothesize that the condition results from a combination of primary opioid receptor-mediated changes in neuronal metabolism that are exacerbated by secondary hypoxic insult. If recognized promptly, this syndrome can be treated with emergent neurosurgical intervention with good clinical outcomes. These cases also illustrate the unintended consequences and innocent victims of the spiraling prescription opioid epidemic, which will likely increase in prevalence. Recognition of this syndrome by clinicians is thus critical.

## Introduction

The United States (US) is in the midst of a nation-wide drug overdose epidemic (1). Data from the Centers for Disease Control and Prevention reveals that drug overdoses increased by close to threefold between 1999 and 2014. In 2015, the death toll from drug overdose in the US was 52,404. Over 60% of these deaths were opioid-related (1). It has been well-established that legal opioid prescription patterns in the US correlate with opioid-related deaths (2). Nearly 50% of all opioid-related deaths in the US involve a prescription opioid (3), with methadone, oxycodone, morphine, and hydrocodone, the most commonly abused drugs in this category (4).

Opioid-induced neurotoxicity is a multifactorial syndrome, with a wide spectrum of symptoms, including confusion, hallucinations, delirium, and seizures, which have been well described in adults (5). In contrast, the effects of opioid intoxication in children are poorly understood and have been described in only a handful of cases (6). A better understanding of the clinical presentation, radiographic findings, and pathophysiological mechanisms of accidental prescription opioid intoxication in children is important given the persistently elevated opioid prescription rates across the US (7).

Herein, we present two recent cases of toddlers who ingested prescription opioids resulting in acute neurological decline, with associated malignant cerebellar edema followed by hydrocephalus, tonsillar herniation, and the need for emergent neurosurgical intervention for life-saving treatment. Together with other recent reports depicting a nearly identical clinical-radiographic presentation, these cases suggest the existence of an uncommon yet severe syndrome of malignant cerebellar edema of probable multifactorial origin. An increased awareness of this condition is of paramount importance given the rising prevalence of accidental prescription opioid intoxications among the pediatric population in the context of the ongoing adult opioid epidemic (8).

## Background

All procedures in this study comply with Yale University’s Human Investigation Committee and Human Research Protection Program. Oral and written informed consent was obtained from the parents of both patients whose cases are herein reported.

### Case 1

A previously healthy 10-month-old female was found by her mother after waking in the morning to have slow, labored breathing. She was unresponsive to voice and tactile stimulation. This prompted a call to 911. On arrival, emergency medical services personnel noted that the infant was non-arousable, bradypneic, and stridorous, with a blood oxygen saturation of 80% on room air. She was transported to the emergency department of a regional hospital, where orotracheal intubation was immediately performed. Her blood pressure on arrival was 93/53 mmHg (mean arterial pressure 66 mmHg). Neurologic examination revealed 1 mm hyporeactive pupils bilaterally, global hypertonia, and hyperreflexia. A non-contrast computed tomography (CT) scan of the head was obtained. Arterial blood gas demonstrated a combined metabolic and respiratory acidosis (pH 7.15, PaCO_2_ 29, PaO_2_ 103, ${\text{HCO}}_{3}^{-}$ 10). Additional laboratory evaluation revealed mild transaminitis (aspartate aminotransferase 100 IU/L, normal = 8–50 IU/L; alanine aminotransferase 55 IU/L, normal = 7–45 IU/L) and leukocytosis (31,000/mm^3^, normal = 6,000–11,000/mm^3^). A broad differential diagnosis of respiratory failure of unknown origin was proposed, and treatment was pursued for possible allergic reaction or meningitis with two doses of epinephrine (0.085 mg), methylprednisolone (2 mg/kg), diphenhydramine (2 mg/kg), and a meningitic dose of ceftriaxone. The patient was then transferred to our tertiary care center.

**Figure 1:**
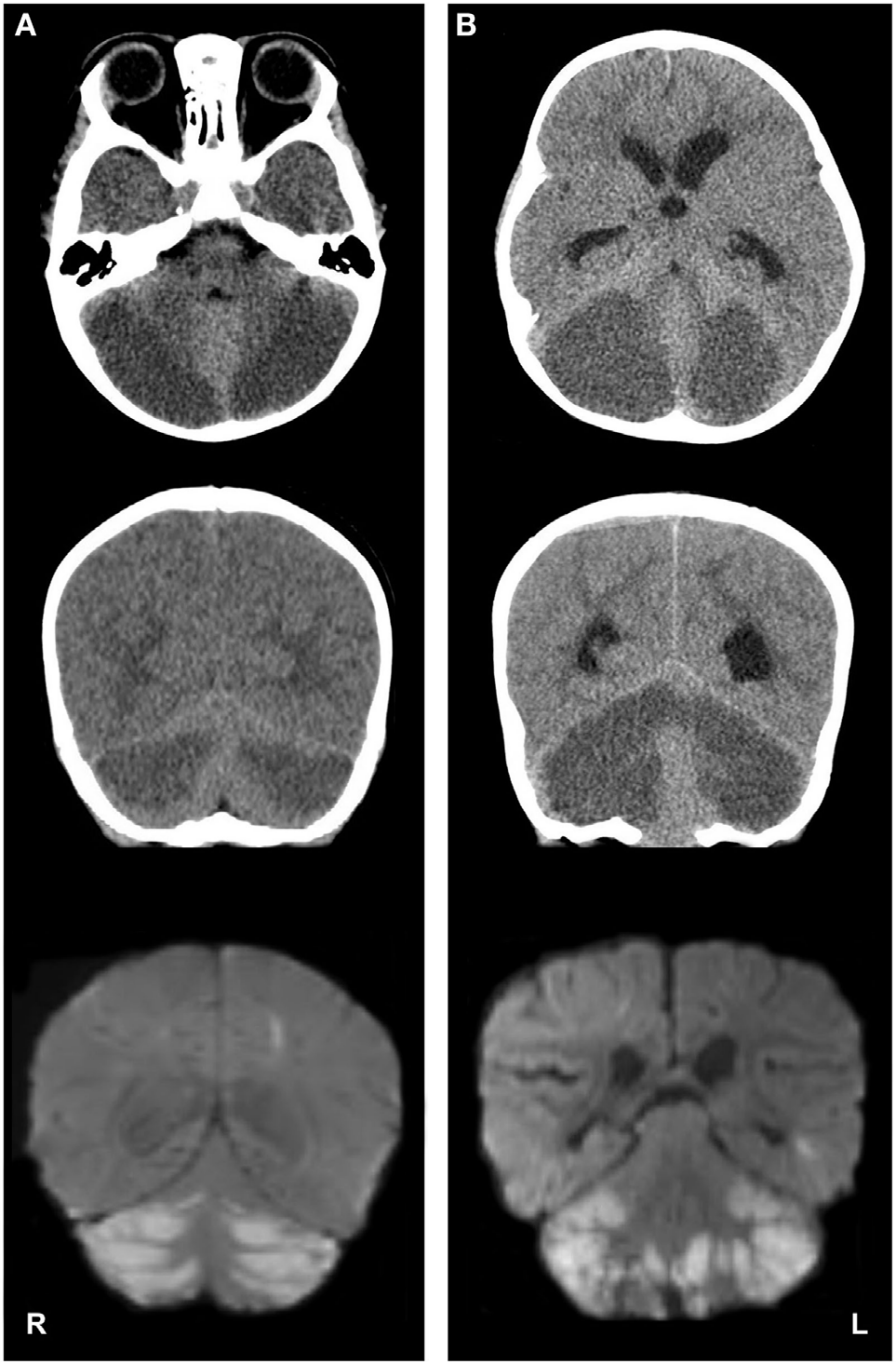
Imaging studies depicting cerebellar edema and restricted water diffusion after accidental opioid overdose in two infants. Panel **(A)**, case 1; panel **(B)**, case 2. Top row: axial computed tomography (CT) images show severe, bilateral cerebellar hypoattenuation, with ventral displacement of the cerebellar vermis and compression of the fourth ventricles. Middle row: coronal CT images demonstrate bilateral cerebellar hypoattenuation and upward displacement of the tectal plate. Bottom row: coronal magnetic resonance diffusion-weighted imaging (DWI) depicts bilateral, symmetric cerebellar diffusion restriction [note: DWI images on panel **(B)** are postoperative].

On admission, workup was expanded with a full toxicology panel, which was positive for oxycodone and its metabolite, oxymorphone. Of note, elevated transaminases raised concern for acetaminophen toxicity; however this was ruled out by a negative result on the toxicology panel. A diagnosis of respiratory failure secondary to opiate ingestion and overdose was established. A total of four doses of naloxone (0.1 mg/kg i.v. push each) were administered with improvement in level of consciousness and reduction of ventilator support that allowed extubation. Continuous naloxone infusion was administered for 5 h thereafter. Results of initial neuroimaging were reviewed and revealed extensive symmetric bilateral cerebellar hypoattenuation (Figure 1A). The patient was admitted to the pediatric intensive care unit and was placed under close observation with frequent neurological exams.

On the third day of hospitalization, she became obtunded, with hyporeactive pupillary reflexes, hypertonia, hypertension, and ocular bobbing. Emergent magnetic resonance imaging (MRI) of the brain was obtained. The MRI demonstrated a pattern of symmetric restricted diffusion in the cerebellar hemispheres (Figure 1A), right parietal lobule, the heads of the caudate nuclei bilaterally and the right putamen. Additionally, hydrocephalus, elevation of the tectum, cerebellar tonsillar herniation, and an area of increased susceptibility weighted imaging signal in the left perirolandic area were present which suggested hemorrhagic transformation of an infarct.

The patient was taken emergently to the operating room for placement of an external ventricular drain, suboccipital craniectomy, and a C1 laminectomy. A significant portion of the edematous and ischemic cerebellar hemispheres and tonsils were resected bilaterally, thereby decompressing the posterior fossa and cervicomedullary junction.

A postoperative magnetic resonance angiography (MRA) of the head and neck showed normal anterior and posterior circulations. A full coagulopathy workup and echocardiography were unremarkable. No other visceral or musculoskeletal injuries were noted, and an investigation by the child abuse team provided no evidence for physical abuse. After an uneventful postoperative course, the patient was discharged on postoperative day 11.

Follow-up at 33 months of age (23 months after presentation) demonstrated an active, engaged child who spoke in full sentences. She was able to ambulate, jump, and climb stairs with alternating feet but had mild spasticity and internal rotation of her left leg with gait and occasional dystonic posturing of the left upper extremity.

### Case 2

A 25-month-old male with a history of mild reactive airway disease and sickle cell trait was noted by his mother to be breathing slowly and erratically 2 h after being laid down for a nap. Emergency medical services were called and paramedics encountered a pulseless, apneic patient. Cardiopulmonary resuscitation was immediately initiated. Return of spontaneous circulation was achieved in the ambulance on route to our institution’s emergency department.

On arrival, physical examination revealed an afebrile toddler, unresponsive to voice or noxious stimulation with agonal respirations, a blood oxygen saturation of 93% with bag-mask-assisted ventilation, and a blood pressure of 99/65 mmHg (mean arterial pressure 76 mmHg). Neurologic examination demonstrated 3 mm, non-reactive pupils and global hyperreflexia. Orotracheal intubation was performed emergently. Initial laboratory evaluation revealed normal electrolytes (Na^+^ 138 mEq/L, K^+^ 4.9 mEq/L, Cl^−^ 104 mEq/L, Ionized Ca^2+^ 5.2 mEq/L), leukocyte count (11,100/mm^3^), venous blood gases (pH 7.37, PvCO_2_ 44, PvO_2_ 47, ${\text{HCO}}_{3}^{-}$ 25.7), and serum lactate (0.9 mmol/L). Upon further interrogation, the patient’s mother reported the presence of prescription opiates (extended release morphine) in the household, which she uses for pain control secondary to frequent sickle cell anemia crises. A total of two doses of naloxone (0.1 mg/kg i.v. push each) were administered, after which the patient regained pupillary reactivity and began moving all four extremities spontaneously. A urine toxicology panel was obtained, which was positive for morphine.

A non-contrast head CT was obtained (Figure 1B), which revealed acute hydrocephalus and extensive, symmetric bilateral cerebellar hemispheric hypoattenuation consistent with severe cerebellar edema that resulted in compression of the fourth ventricle and displacement of the midbrain and pons superiorly. Cervicomedullary junction compression secondary to cerebellar tonsillar herniation was also noted. Neurosurgery was consulted and the patient was emergently taken to the operating room for the placement of an external ventricular drain, suboccipital craniectomy, and a C1 laminectomy. A significant portion of the edematous and ischemic cerebellar hemispheres and tonsils were resected bilaterally, thereby decompressing the posterior fossa and cervicomedullary junction.

A brain MRI obtained on postoperative day 1 demonstrated cerebellar and cervicomedullary junction decompression, with extensive restricted diffusion in the remaining cerebellar parenchyma (Figure 1B). Multifocal areas of diffusion restriction were also noted bilaterally in the anterior cerebral artery-middle cerebral artery and middle cerebral artery-posterior cerebral artery watershed territories involving portions of the frontal, occipital, and temporal lobe white matter. An MRA/MR venogram demonstrated no craniocervical or intracranial arterial abnormalities or evidence of cortical or dural venous sinus thrombosis. The patient was extubated on postoperative day 5. Assessment at postoperative day 18 demonstrated an easily arousable child, with spontaneous eye opening, equally round and reactive pupils, and with purposeful movements of all four extremities.

Outpatient follow-up at 29 months of age (3 months after presentation) demonstrated an active child speaking in full sentences. He was able to ambulate independently, albeit with a slightly widened base of support, and mild left lower extremity spasticity which did not limit his ability to climb or descend stairs with rail support.

## Discussion

We describe two toddlers with malignant cerebellar edema after accidental prescription opioid ingestion. Both children developed acute neurological decline, hydrocephalus, and tonsillar herniation that required emergent cerebrospinal fluid diversion and surgical decompression of their posterior fosse. In both instances, patients had good clinical outcomes though with some deficits considering the life-threatening nature of their presentations. To our knowledge, there are only two other reported cases of children requiring surgical posterior fossa decompression for malignant cerebellar edema after accidental prescription opioid ingestion (9, 10). Both cases had nearly identical clinical courses and radiographic findings when compared to our patients. Together, these four cases suggest the existence of an uncommon but severe syndrome of predominantly cerebellar malignant edema. If recognized promptly, this syndrome can be treated with emergent neurosurgical intervention with good clinical outcomes. These cases also illustrate the “trickle-down” effect of the current prescription opioid epidemic, which will likely increase the prevalence of this syndrome.

Direct neurotoxicity is the hallmark of multiple narcotic as well as non-narcotic drugs and toxins. Several molecules with neurotoxic effects exhibit predilection for white matter, which over the years has received the blanket term “toxic leukoencephalopathy” (11). This white matter-predominant pattern of injury has been reported after exposure to various other non-opioid substances including toluene, cocaine, methamphetamine, and chemotherapeutic agents such as 5-fluorouracil and methotrexate, among others (12–17).

The neurologic sequelae of opiate intoxication and overdose are multiple, including brain injury from primary neurotoxicity and secondary hypoxia/anoxia (18–20). Numerous reports and anecdotal descriptions of the direct neurotoxic effects of opiates are present in the medical literature (18, 21, 22). These effects are well documented in adults. Clinically, presentations are heterogeneous and include a wide range of symptoms from decreased alertness to more severe obtundation, delirium, and seizures (5, 23). Histopathologic examination of white matter samples collected from cases of opioid-induced leukoencephalopathy reveal spongiform degeneration, oligodendroglial vacuolization, and fluid entrapment between myelin lamellae, without demyelination (21). Radiologically, on head CT, a symmetric, diffuse hypoattenuation of both supratentorial and infratentorial white matter is often recognized. On MRI, diffuse cortical swelling and cerebellar hyperintensity on T2-weighted and diffusion-weighted imaging (DWI) are present. Interestingly, in adult cases of inhalation exposure to non-prescription opioids, supra and infratentorial leukoencephalopathic changes, hallmarked by cerebellitis is relatively common, and has been labeled “chasing the dragon” syndrome (24).

In contrast, there is a paucity of information regarding the neurological effects of prescription opioid overdose in children. Our comprehensive review of the literature revealed 8 additional cases of acute pediatric opioid-induced leukoencephalopathy with cerebellar edema (Table 1) (9, 10, 25–30). Patients were toddlers, who accidentally consumed their caregiver’s prescription opiates orally with the exception of one case in which administration occurred through a transdermal fentanyl patch (27). In a single instance, the exposure was to raw opium with no mention of the route of administration (28). All children developed obtundation, often severe enough to require orotracheal intubation. Abnormal neurologic examination findings were universally described, and included altered consciousness ranging from obtundation to coma, bradypnea, miotic or hyporesponsive pupils, hyperreflexia and/or hypertonia, and ataxia. Interestingly, in all reported cases, there are several common neuroradiologic characteristics. Often, the first study obtained in the emergency department was a non-contrast head CT, which revealed bilateral and symmetric cerebellar hypoattenuation, with a variable element of cisternal effacement and hydrocephalus, dependent on the magnitude of mass effect exerted by parenchymal edema. On MRI, cerebellar diffusion restriction on DWI, and hyperintensity on T2-weighted and fluid attenuation inversion recovery sequences were frequently described (9, 10, 25, 28). Restricted diffusion, albeit most marked in the cerebellum, was also present in watershed areas of the deep white matter, where circulatory redundancy between the anterior and posterior circulation is minimal. This draws an interesting parallel between opioid-induced acute cerebellar edema in toddlers and “chasing the dragon” syndrome in adults.

**Table 1:** Reported cases of pediatric opioid-induced malignant cerebellar edema.

#	Age	Sex	Opiate	Treatment	Neurologic outcome	Country	Year	Reference
1	3 years	M	Methadone	Naloxone; EVD; IV methylprednisolone	Intact at 6-weeks follow-up	Portugal	2006	Anselmo et al. (25)

2	22 months	M	Methadone	Naloxone; observation	Death at a few weeks post intoxication	USA	2008	Riascos et al. (30)

3	3 years	F	Methadone	Naloxone; EVD; suboccipital craniectomy	Cognitively intact, mild spasticity, mild cortical visual impairment at 4-month follow-up	England	2008	Mills et al. (9)

4	19 months	F	Fentanyl	Observation	Intact at 3 days post intoxication	USA	2011	Foy et al. (27)

5	3 years	M	Methadone	Observation	Death at 8 h after admission	Portugal	2012	Mendes-dos-Santos et al (29)

6	^a^	^a^	Methadone	EVD	^a^	Iran	2014	Bazmamoun et al. (26)

7	2 years	F	Morphine	Naloxone; EVD; suboccipital craniectomy	Short sentences, non-ambulatory, light perception in both eyes at 18-month follow-up	USA	2015	Reisner et al. (10)

8	2 years	F	Opium	IV methylprednisolone	Intact at 2-month follow-up	Iran	2016	Hosseini and Nikkhah (28)

9	10 months	F	Oxycodone	Naloxone; EVD; suboccipital craniectomy	Cognitively intact, ambulatory, mild spasticity at 23-month follow-up	USA	2017	Duran et al. (this study)

10	2 years	M	Morphine	Naloxone; EVD; suboccipital craniectomy	Cognitively intact, ambulatory, mild left lower extremity spasticity at 3-month follow-up	USA	2017	Duran et al. (this study)

*Age, sex, opioid consumed, and treatment administered in each reported case of opioid-induced cerebellar edema*.

*EVD, external ventricular drain, IV, intravenous, USA, United States of America, POD, postoperative day*.

*^a^Information not reported*.

Typical areas of selective brain vulnerability to insult vary with age and mechanism. In the pediatric population, these areas are characterized by elevated metabolic activity, such as gray matter, or areas of active myelination (31). In neonates, infants, and toddlers, cerebellar injury secondary to hypoxia or neuroinflammation is uncommon, especially in the setting of hypoxic insult (32). During times of mild to moderate hypoxic–ischemic insult, autoregulatory mechanisms are able maintain perfusion to vital structures such as the brainstem, thalami, basal ganglia, hippocampi, and cerebellum by shunting blood from less metabolically active structures, such as the cerebral cortex and white matter (32). In severe hypoxic–ischemic encephalopathy, the cerebellum is often the last structure affected, leading it to appear bright on head CT (“bright cerebellar sign”). The pattern of primary hypoxic–ischemic encephalopathy is thus different from our cases in which the cerebellum was most severely affected. Furthermore, non-accidental injury was considered in both of our cases but was unlikely given the lack of typical imaging or physical findings to support this diagnosis like signs of external trauma, subdural hemorrhage, or hemorrhage in the high cervical cord or other cervical spine injury (33, 34).

Predominantly cerebellar injury, along with more discrete areas of supratentorial leukoencephalopathy appears to be a distinct pattern as a response to opioid neurotoxicity in the pediatric population. Of note, the distribution and predominance of opiate receptors differs between the adult and developing human cerebellum (35), providing a plausible explanation for the differential presentation of opioid-induce neurotoxicity observed across age groups. Unfortunately, no histopathologic examination of excised cerebellar tissue has been performed in pediatric patients, including the two cases we present above.

The striking resemblance in presentation, patient characteristics, neuroradiologic findings, and triggering mechanism between these cases leads us to believe we are facing a variant of opioid-induced encephalopathy, primarily affecting the pediatric population. We hypothesize this condition is the result of a multifactorial insult primarily affecting white matter, most striking in the cerebellum, due to the combined influence of direct opioid receptor-mediated phenomena altering neuronal and/or glial metabolism, aggravated by the influence of the anoxic injury secondary to respiratory depression or arrest in the context of opioid overdose. Formal pathologic examination of excised tissue in these cases will aid in determining the magnitude and type of acute cellular response in similar cases, allowing to better adjudicate a differential burden of responsibility to primary, opioid-induced neuroinflammation versus hypoxia/anoxia in cases of pediatric opioid-induced neurotoxicity.

Ideal management strategies for pediatric opioid-induced malignant cerebellar edema are yet to be defined, as the natural history and progression of this condition remains poorly described. In acute cerebellar edema of other etiologies (frequently secondary to inflammation—i.e., cerebellitis), there is marked clinical variability; the condition can have a relatively benign and self-limited progression, or result in fulminant swelling leading to tonsillar herniation and death (36). Careful neurologic evaluation and early neuroimaging should be routinely performed for pediatric patients in the setting of opioid intoxication or overdose. Medical management for acute cerebellitis of other etiologies, and in specific cases of opioid-induced pediatric acute malignant cerebellar edema, focused on amelioration of cerebral and cerebellar inflammation with steroids has been attempted (25, 28, 37). The role of surgical intervention in cases of severe pediatric cerebellitis has been previously described (38). Neurosurgical intervention, aimed at posterior fossa and cervicomedullary junction decompression should be considered in cases in which herniation is imminent or in progress and unlikely to be ameliorated with medical therapy alone.

Pediatric opioid overdose remains a relatively rare occurrence in the US (39). However, epidemiologic data and published reports suggest a disturbingly increasing trend, which mirrors the sharp increase in opioid overdose cases in adults (40). As prescription and illegal opiates continue to make their way to households with children, the risk of exposure will continue to increase.

## Concluding Remarks

In summary, we describe two cases of pediatric acute toxic cerebellar edema associated with oral prescription opiate ingestion and incorporate these into a comprehensive review of existing literature. Opioid-induced malignant cerebellar edema should be included in the differential diagnosis when a pediatric patient presents with signs and symptoms of opiate intoxication and an abnormal neurologic examination. Raising awareness of this condition is of paramount importance, given the increasing prevalence of pediatric opioid intoxication. Future directions should include microscopic examination of excised cerebellar tissue in this context, to clarify the pathophysiology of this emerging condition.

## Ethics Statement

All procedures in this study comply with Yale University’s Human Investigation Committee (HIC) and Human Research Protection Program. Oral and written informed consent was obtained from the parents of both patients whose cases are herein reported.

## Author Contributions

DD and RM were the principal authors of this manuscript with guidance from both KK and LB. JM, JK, CF, AS, and YY were significantly involved in the research portion of the paper and contributed to portions of the write up as well. The paper was reviewed, revised, and critically analyzed by GS, MD, and KK for imaging interpretation in the case of GS and the surgical content by MD and KK who performed the surgical procedures on case 1 and case 2, respectively. All throughout the process, LB was instrumental in her guidance and critical evaluation of the manuscript for content and accuracy. LB was the principle editor of the pediatric neurology aspect, RM of the neuroradiology aspect, and KK from the neurosurgical aspect. LB and RM were the principal editors throughout the process and were under the guidance of KK.

## Conflict of Interest Statement

The authors declare that the research was conducted in the absence of any commercial or financial relationships that could be construed as a potential conflict of interest.
